# Efficient production and secretion of pyruvate from *Halomonas* sp. KM-1 under aerobic conditions

**DOI:** 10.1186/s13568-016-0195-y

**Published:** 2016-03-18

**Authors:** Yoshikazu Kawata, Taku Nishimura, Isao Matsushita, Jun Tsubota

**Affiliations:** Biomedical Research Institute, National Institute of Advanced Industrial Science and Technology (AIST), 1-8-31 Midorigaoka, Ikeda, Osaka, 563-8577 Japan; Energy Technology Laboratories, OSAKA GAS Co., Ltd., 6-19-9 Torishima, Konohana-ku, Osaka, 554-0051 Japan

**Keywords:** *Halomonas*, Pyruvate, Poly-(*R*)-3-hydroxybutyric acid, Nitrate

## Abstract

The alkaliphilic, halophilic bacterium *Halomonas* sp. KM-1 can utilize both hexose and pentose sugars for the intracellular storage of bioplastic poly-(*R*)-3-hydroxybutyric acid (PHB) under aerobic conditions. In this study, we investigated the effects of the sodium nitrate concentration on PHB accumulation in the KM-1 strain. Unexpectedly, we observed the secretion of pyruvate, a central intermediate in carbon- and energy-metabolism processes in all organisms; therefore, pyruvate is widely used as a starting material in the industrial biosynthesis of pharmaceuticals and is employed for the production of crop-protection agents, polymers, cosmetics, and food additives. We then further analyzed pyruvate productivity following changes in culture temperature and the buffer concentration. In 48-h batch-cultivation experiments, we found that wild-type *Halomonas* sp. KM-1 secreted 63.3 g/L pyruvate at a rate of 1.32 g/(L·h), comparable to the results of former studies using mutant and recombinant microorganisms. Thus, these data provided important insights into the production of pyruvate using this novel strain.

## Introduction

Pyruvate is a central intermediate in carbon and energy metabolism in all organisms; therefore, pyruvate is widely used as a starting material in the industrial biosynthesis of pharmaceuticals and is employed for production of crop-protection agents, polymers, cosmetics, and food additives (Li et al. 2001). Chemical production of pyruvate has been achieved by dehydration and decarboxylation of tartrate (Howard and Fraser 1932). However, this process is not cost-effective (Li et al. 2001); hence, biotechnological pyruvate production has attracted attention as a potential alternative method of pyruvate synthesis. To date, the successful biotechnological production of pyruvate from glucose has primarily been achieved using bacteria, such as *Escherichia coli* (Causey et al. 2004; Yokota et al. 1994; Zhu et al. 2008) and *Corynebacterium glutamicum* (Wieschalka et al. 2012), and yeasts, such as *Saccharomyces cerevisiae* (van Maris et al. 2004) and *Torulopsis glabrata* (Liu et al. 2007; Miyata and Yonehara 1999).

Halophilic bacteria are utilized in industrial chemical-production processes owing to their unique properties, such as contamination-free culture conditions and a tolerance for high substrate concentrations (Quillaguamán et al. 2010; Yin et al. 2015). In addition, alkaliphilic bacteria are utilized to produce pure organic acids (Calabia et al. 2011; Yokaryo and Tokiwa 2014). Therefore, alkaliphilic and halophilic bacteria are appropriate candidates for industrial production of pure organic acids.

The alkaliphilic, halophilic bacterium *Halomonas* sp. KM-1 was recently isolated and found to store bioplastic poly-(*R*)-3-hydroxybutyric acid (PHB) intracellularly under aerobic conditions (Kawata and Aiba 2010). In addition, the KM-1 strain secretes pure (*R*)-3-hydroxybutyric acid ([*R*]-3-HB) under microaerobic conditions (Kawata et al. 2012a). The KM-1 strain has potential for use in industrial fermentation applications, with some specific advantages over other microorganisms, particularly for the industrial-scale production of organic acids. First, sterilization of culture medium is not required to eliminate contamination by other bacteria because the KM-1 strain can be cultured in modified SOT medium that is alkaline (pH 9.4), with moderate salinity. Moreover, modified SOT medium is an inexpensive, simple, and chemically defined medium. Second, biotechnological production using the KM-1 strain is not subject to strict safety regulations because it is neither pathogenic nor recombinant. Third, the KM-1 strain can utilize a variety of carbon sources; surprisingly the KM-1 strain can utilize C6 and C5 sugars in parallel, without glucose catabolite repression (Kawata et al. 2012a, 2013). In addition, this strain has already been employed to produce PHB from biodiesel waste glycerol and saccharified wood (Kawata and Aiba 2010; Kawata et al. 2013). Finally, because the KM-1 strain is tolerant of moderately high osmolality, a high concentration of the substrate, e.g., 20 % glucose, may be used to facilitate high productivity of pure organic acids. Thus, the KM-1 strain has been evaluated for potential applications in the industrial-scale production of PHB and (*R*)-3-HB (Kawata et al. 2012a, 2013, 2014).

In the metabolic pathway of PHB synthesis, the carbon source (glucose) is converted to phosphoenolpyruvate (PEP), and PEP is then converted to pyruvate by pyruvate kinase (PK). Pyruvate is decarboxylated by the pyruvate dehydrogenase complex (PDC) to produce acetyl-CoA, which is then used for subsequent reactions. When the sodium nitrate concentration is increased, the entire metabolic pathway of PHB synthesis would likely be activated; thus, higher PHB productivity would be expected. Interestingly, however, in our preliminary work, we found that increased sodium nitrate concentrations resulted in reduced PHB productivity and enhanced pyruvate secretion. Therefore, in this study, we further explored this observation and investigated changes in pyruvate production with different nitrate concentrations, culture temperatures, and buffer salinities in simple batch cultivations of the KM-1 strain under aerobic conditions.

## Materials and methods

### Culture conditions

In this study, we used *Halomonas* sp. KM-1, which was deposited at the National Institution of Technology Evaluation as FERM BP-10995 (Kawata and Aiba 2010). KM-1 cells were cultivated using 20 mL modified unsterilized SOT medium supplemented with 20 % (w/v) glucose, with initial sodium hydrogen carbonate and sodium carbonate concentrations of 0.2 M (pH 9.4), in 200-mL Erlenmeyer flasks with rotational shaking at 200 rpm at 33 °C (Kawata et al. 2012a).

Optimal pyruvate production conditions were investigated by varying the sodium nitrate concentration (15.0, 20.0, 25.0, and 30.0 g/L), culture temperature (33, 37, 40, and 43 °C), and sodium hydrogen carbonate and sodium carbonate concentration (0.2, 0.3, 0.4, 0.5, and 0.6 M; maintaining pH 9.4). All experiments were performed in triplicate.

### Analysis of PHB, glucose pyruvate, and pH levels

To measure PHB content, samples were collected every 12 h, unless otherwise stated. PHB contents were analyzed by gas chromatography (Kawata et al. 2012a; Monteil-Rivera et al. 2007) using a commercial PHB sample (Fluka, Buchs, Switzerland) used as a standard. Glucose and pyruvate contents were analyzed by high-performance liquid chromatography (HPLC), as described by the manufacturer (Aminex HPX-87H; Bio-Rad, Tokyo, Japan) with commercially available, pure samples of d-glucose and pyruvate (Wako, Osaka, Japan) used as standards. The pH levels of the different media were analyzed using a portable D-71 pH meter with a Micro ToupH electrode 9618S-10D (Horiba, Kyoto, Japan).

## Results

### Effects of changing the sodium nitrate concentration on the production of pyruvate and PHB by KM-1 cells

**Fig. 1:**
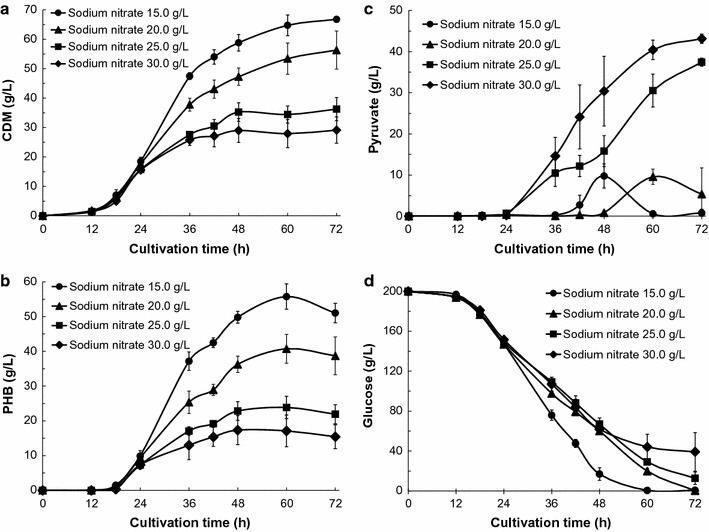
Pyruvate and PHB production by *Halomonas* sp. KM-1 in the presence of different concentrations of sodium nitrate. KM-1 cells were cultured under aerobic conditions (agitation speed: 200 rpm) at 33 °C. The medium was composed of modified SOT medium, and 15.0 (*circles*), 20.0 (*triangles*), 25.0 (*squares*), or 30.0 g/L (*diamonds*) sodium nitrate. **a** Cell dry mass (CDM), **b** intracellular PHB, **c** pyruvate in the medium, and **d** glucose in the medium were analyzed. The data shown represent the mean ± SD of three independent experiments

To increase PHB productivity, we examined the effects of increasing sodium nitrate concentrations (15.0, 20.0, 25.0, and 30.0 g/L) in simple batch cultivations of the KM-1 strain under aerobic conditions. The amounts of cell dry mass (CDM) and PHB produced by the KM-1 strain over time are shown in Fig. 1a, b. Maximal CDM production were observed after 72 h of aerobic cultivation in all samples, whereas maximal PHB production was observed at 60 h. In previous studies, yields of 26.9 and 59.9 g/L PHB and 37.2 and 73.8 g/L CDM were obtained after 36 or 60 h of cultivation, respectively, with the KM-1 strain under aerobic conditions in simple batch cultivation in the presence of 12.5 or 20.0 g/L sodium nitrate, respectively, as the sole nitrogen source (Kawata et al. 2013, 2014). Increasing the concentration of sodium nitrate in batch culture medium was expected to increase the production of both CDM and PHB; however, the production of CDM and PHB decreased compared with the levels observed in previous studies (Fig. 1a, b) (Kawata et al. 2013, 2014).

In the same experiment, analysis of glucose contents in the medium by HPLC revealed that glucose was consumed at a constant rate until 60 h of cultivation, as expected based on the PHB-production levels observed (Fig. 1b, d). In particular, for samples supplemented with 20.0, 25.0, and 30.0 g/L sodium nitrate, the speed of glucose consumption was generally similar, whereas the maximum PHB production decreased as the sodium nitrate concentration increased (Fig. 1b, d). Surprisingly, we also observed an increase in pyruvate concentrations in the medium as the amount of sodium nitrate increased (Fig. 1c). Thus, we expected that some of the consumed glucose was converted to pyruvate. We selected 30.0 g/L sodium nitrate for use in further experiments.

### Effects of changing the culture temperature on pyruvate production by KM-1 cells

**Fig. 2:**
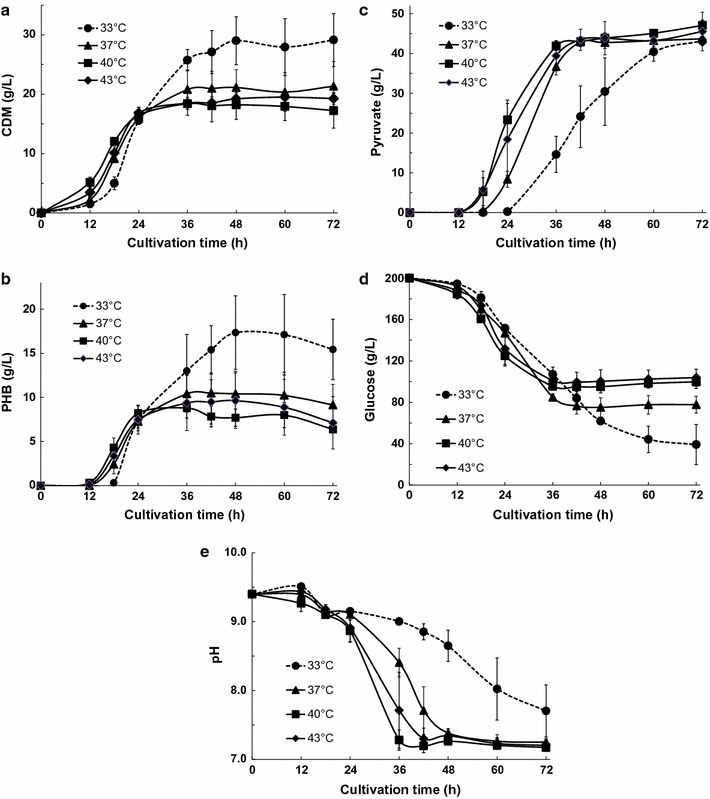
Pyruvate and PHB production by *Halomonas* sp. KM-1 under different culture temperatures. The medium was composed of modified SOT medium containing 30.0 g/L sodium nitrate. KM-1 cells were cultured under aerobic conditions (agitation speed: 200 rpm) at 33 °C (*circles*; *dotted line* in Fig. 1), 37 °C (*triangles*), 40 °C (*squares*), or 43 °C (*diamonds*). **a** Cell dry mass (CDM), **b** intracellular PHB, **c** pyruvate in the medium, **d** glucose in the medium, and **e** pH of the medium were analyzed. The data shown represent the mean ± SD of three independent experiments

Next, we examined whether changes in the culture temperature affected pyruvate productivity in simple batch cultivations of KM-1 cells under aerobic conditions. Changes in CDM, PHB, and pyruvate production; glucose consumption; and the pH of the medium observed over time with KM-1 cells are shown in Fig. 2a–e. For samples cultured at 33, 37, 40, and 43 °C, the amounts of CDM and PHB were similar at 24 h; however, samples cultured at 40 and 43 °C consumed more glucose than samples cultured at 33 and 37 °C (Fig. 2a, b, d). The differences in glucose consumption between both groups were mostly related to changes in pyruvate contents, and samples cultured at 37, 40, and 43 °C exhibited the highest levels of pyruvate production at 36 h (Fig. 2c).

### Effects of changing the sodium bicarbonate buffer concentration on pyruvate production by KM-1 cells

**Fig. 3:**
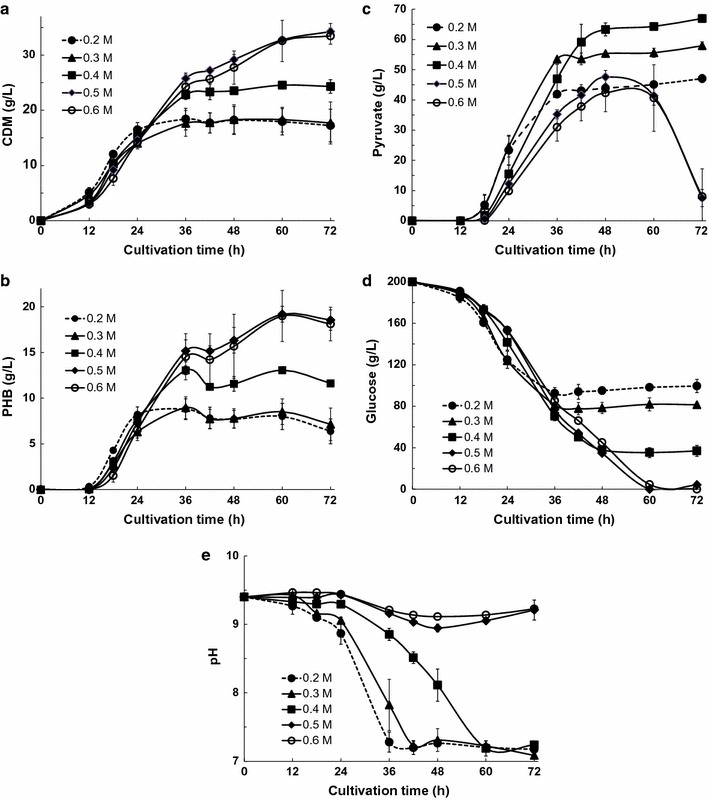
Pyruvate and PHB production by *Halomonas* sp. KM-1 in the presence of different concentrations of sodium bicarbonate buffer KM-1 cells were cultured under aerobic conditions (agitation speed: 200 rpm) at 40 °C. The medium was composed of modified SOT medium containing 30.0 g/L sodium nitrate with different concentrations of sodium bicarbonate buffer, i.e., 0.2 M (*closed circles*; *dotted line* in Fig. 2), 0.3 M (*triangles*), 0.4 M (*squares*), 0.5 M (*diamonds*), or 0.6 M (*open circles*). **a** Cell dry mass (CDM), **b** intracellular PHB, **c** pyruvate in the medium, **d** glucose in the medium, and **e** pH of the medium were analyzed. The data shown represent the mean ± SD of three independent experiments

Liu et al. (2007) reported an enhancement of pyruvate production by the osmotic-tolerant mutant *Candida glabrata* (*Torulopsis glabrata*) RS23. The KM-1 strain has a relatively high tolerance for salinity until the NaCl concentration reaches 10 % (w/v; 1.7 M) (Kawata et al. 2012b). Therefore, we examined the optimum salinity for pyruvate production by altering the concentration of sodium bicarbonate buffer (0.2, 0.3, 0.4, 0.5, and 0.6 M) during incubation at 40 °C using 30.0 g/L sodium nitrate in simple batch cultivations with the KM-1 strain under aerobic conditions. Changes in CDM, PHB, and pyruvate production; glucose consumption; and changes in the pH observed over time with KM-1 cells are shown in Fig. 3a–e.

The amounts of CDM and PHB measured were similar up through 24 h. Subsequent increases in CDM and PHB levels were blocked in samples with 0.2, 0.3, or 0.4 M buffer at 36 h. However, for samples cultured in 0.5 or 0.6 M buffer, the CDM and PHB levels continued to increase until 60 h (Fig. 3a, b). Similarly, glucose consumption was suppressed in samples cultured in 0.2 or 0.3 M buffer after 36 h and in samples cultured in 0.4 M buffer after 48 h; glucose consumption was not halted in samples cultured in 0.5 or 0.6 M buffer until 72 h (Fig. 3d). Because the pyruvate concentration exceeded more than the individual buffer concentration, the pH in the medium decreased to below 8.0, after which the pyruvate concentration did not increase (Fig. 3c, e). For samples cultured with 0.5 or 0.6 M buffer, after the consumption of glucose, the increase in the pyruvate concentration was blocked after 60 h (Fig. 3c, d), suggesting that the secreted pyruvate may have been taken back up by KM-1 cells. The highest pyruvate levels produced by the KM-1 strain were 63.3 g/L with a productivity of 1.32 g/(L·h) at 48 h and 67.0 g/L with a productivity of 0.93 g/(L·h) at 72 h in simple aerobic batch cultivations, using 0.4 M sodium bicarbonate buffer (Fig. 3c).

Schügerl (2000) suggested that organic acid accumulation may suppress growth and byproduct formation during organic acid fermentation. Moreover, this inhibitory effect may be related to the decrease in pH observed as acids accumulate and subsequent acid-dependent toxicity, particularly at low pH values, at which acids will be preferentially in the undissociated state. The same conditions were employed for pyruvate production in the current study (Fig. 3c). The maximum pyruvate production levels for samples incubated with 0.2, 0.3, or 0.4 M buffer were 47.1, 57.9, and 67.0 g/L, respectively. If the maximum pyruvate production was controlled by the buffer concentration, then pyruvate production in samples containing 0.3 or 0.4 M buffer should have exceeded the observed pyruvate levels (Fig. 3c), as should the pyruvate concentrations observed in samples containing 0.5 or 0.6 M buffer (Fig. 3c). Thus, higher salinity may interfere with the growth of KM-1 cells and pyruvate production.

## Discussion

In this study, pyruvate secretion changes by strain *Halomonas* sp. KM-1 were investigated in the presence of different nitrate concentrations, culture temperatures, and buffer salinities in simple batch cultivations under aerobic conditions. To increase PHB productivity, nitrate concentrations were increased, but PHB productivity was not increased, and pyruvate secretion by KM-1 cells was unexpectedly observed (Fig. 1b, c). These results showed that increased sodium nitrate concentrations promoted pyruvate secretion by the KM-1 strain. Some soil bacteria can use nitrate respiration under aerobic conditions (Carter et al. 1995), and Halomonas type bacterium *Halomonas elongata* can reduce nitrate and grow anaerobically (Vreeland et al. 1980). Based on this phenomenon, the nitrate concentration is associated with pyruvate production; however, the association between pyruvate production and nitrate concentration observed in this study has not been previously described.

The KM-1 strain could be cultured at pH 6.5–10.5, with an optimum pH of 9.4 (Kawata and Aiba 2010; Kawata et al. 2014). When the sodium carbonate buffer concentration in the medium was 0.2 M, pyruvate production resulting in pyruvate concentrations of >0.2 M (17.6 g/L) caused the pH to decrease, blocking additional pyruvate production (Fig. 2e). The maximal pyruvate production was 47.1 g/L (0.53 M) after culturing KM-1 cells at 40 °C for 72 h, when the optimal culture temperature was investigated.

Pyruvate production, both in terms of the quantity and rate, was relatively high in the KM-1 strain compared with that in other microorganisms, particularly when considering that pyruvate production was measured in the wild-type strain, not in mutant or recombinant strains, and that a fed-batch cultivation system was not used (Table 1). Thus, the method presented herein may have advantages over previously described methods, particularly for industrial-scale pyruvate production. As an alkaliphilic and halophilic bacterium, *Halomonas* sp. KM-1 was grown under moderately alkaline conditions (pH 9.4) and with moderate salinity (0.4 M). Moreover, the KM-1 strain did not require sterilization of culture-modified SOT medium, an inexpensive, simple, and chemically defined medium. In addition, the KM-1 strain is neither pathogenic nor recombinant; thus, no strict safety regulations are in place concerning the culture of this strain, and the KM-1 strain can be cultured at simple facilities with a low running cost. In addition, during the industrial production of pyruvate, the levels of organic acids in the medium may interfere with pyruvate purification. In the KM-1 strain, we rarely observed peaks indicative of other organic acids by HPLC analysis (data not shown). Thus, the KM-1 strain may have economic advantages for industrial pyruvate production.

**Table 1:** Pyruvate production by various microorganisms using different procedures

Organism	Carbon source	Procedure	Pyruvate	Production rate	Reference
			(g L^−1^)	(g L^−1^h^−1^)	
*Halomonas* sp. KM-1	Glucose	Wild-type, batch	67.0	0.93	This work (72 h)
*Halomonas* sp. KM-1	Glucose	Wild-type, batch	63.3	1.32	This work (48 h)
*E. coli* ALS1059	Glucose, acetate	Mutant, fed-batch	90.0	2.05	Zhu et al. (2008)
*E. coli* TC44	Glucose	Mutant, fed-batch	52.0	1.21	Causey et al. (2004)
*E. coli* TBLA-1	Glucose	Mutant, batch	31.5	0.98	Yokota et al. (1994)
*S. cerevisiae* TAM	Glucose	Mutant, fed-batch	135.0	1.35	van Maris et al. (2004)
*C. glabrata* (*T. glabrata*) ACII-33	Glucose	Mutant, fed-batch	67.8	1.07	Miyata and Yonehara (1999)
*C. glabrata* (*T. glabrata*) RS23	Glucose	Mutant, continuous culture	94.3	1.18	Liu et al. (2007)
*C. glutamicum* ELB-P	Glucose	Recombinant, fed-batch	44.5	0.49	Wieschalka et al. (2012)

**Fig. 4:**
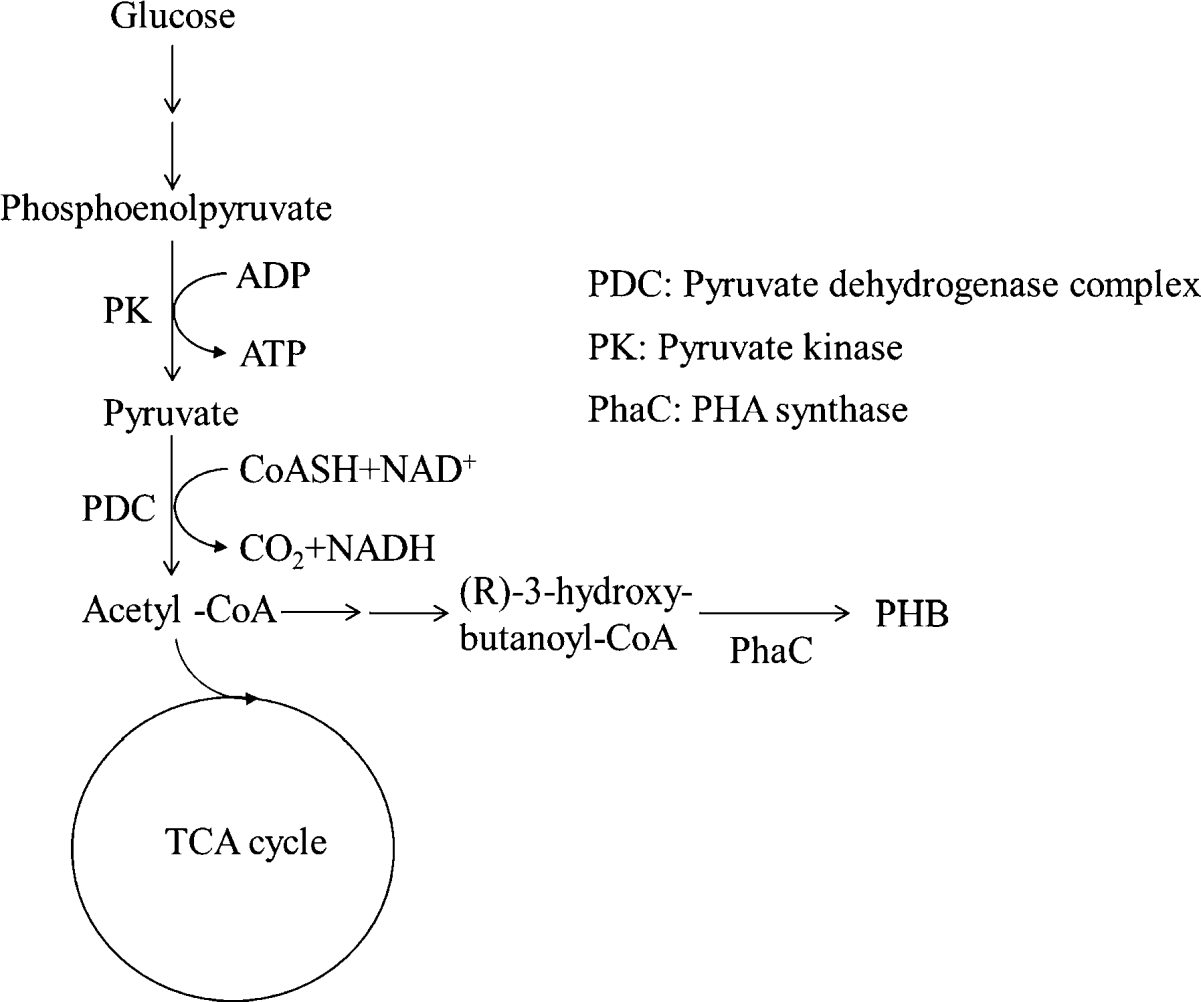
Metabolic pathway of butanoate metabolism in *Halomonas* sp. KM-1

In pyruvate production during glycolysis, glucose is converted to PEP, and PEP is then converted to pyruvate by PK. Next, pyruvate is decarboxylated by PDC, producing acetyl-CoA, which is then used in additional reactions (Fig. 4). Pyruvate is stored when the enzyme activity of PK is higher than that of PDC. Pyruvate production has been studied using different approaches with various microorganisms (Table 1). In other previous studies using recombinant and mutant strains under batch, fed-batch, and continuous culture conditions, using bacteria such as *E. coli* (Causey et al. 2004; Yokota et al. 1994; Zhu et al. 2008) and *C. glutamicum* (Wieschalka et al. 2012), and yeasts, such as *S. cerevisiae* (van Maris et al. 2004) and *T. glabrata* (Liu et al. 2007; Miyata and Yonehara 1999), the targets of recombinations or mutations have primarily been genes involved in pyruvate metabolism, such as PDC, pyruvate: quinone oxidoreductase, and F1-ATPase. In contrast, the KM-1 strain was induced to secrete pyruvate by applying excess sodium nitrate; this type of efficient pyruvate production system has not been well studied. Thus, additional studies are needed to clarify the mechanisms mediating pyruvate secretion under these conditions.

Liu et al. (2007) reported an enhancement of pyruvate production by the osmotic-tolerant mutant *C. glabrata* RS23 strain. The KM-1 strain has a relatively high tolerance for salinity until the NaCl concentration reaches 10 % (w/v; 1.7 M) (Kawata et al. 2012b). Thus, the wild-type KM-1 strain exhibits a natural tolerance of moderately high osmolality; accordingly, a substrate concentration of up to 20 % glucose can be used to facilitate high pyruvate production. Thus, future studies are needed to achieve pyruvate production at an industrial scale using the osmotic-tolerant mutant of the KM-1 strain, which may result in higher pyruvate productivity under high osmotic culture conditions, using a fed-batch culture system.

In this study, the alkaliphilic, halophilic bacterium *Halomonas* sp. KM-1 secreted 63.3 g/L pyruvate with a productivity of 1.32 g/(L·h) by 48 h of aerobic batch cultivation in sodium nitrate- and glucose-rich medium, without sterilization. Pyruvate production was related to the nitrate concentration; this mechanism has not been published previously. Pyruvate production in this study occurred at a relatively high level, and the KM-1 strain offered some advantages, such as contamination-free culture and the capacity for high substrate concentrations. Thus, these results suggest that the KM-1 strain may be a suitable candidate for industrial pyruvate production.
